# Retracted: Melatonin Attenuates Cardiac Ischemia-Reperfusion Injury through Modulation of IP3R-Mediated Mitochondria-ER Contact

**DOI:** 10.1155/2023/9890809

**Published:** 2023-01-25

**Authors:** Oxidative Medicine and Cellular Longevity

*Oxidative Medicine and Cellular Longevity* has retracted the article titled “Melatonin Attenuates Cardiac Ischemia-Reperfusion Injury through Modulation of IP3R-Mediated Mitochondria-ER Contact” [1] due to concerns that the peer review process has been compromised.

Following an investigation conducted by the Hindawi Research Integrity team [2], significant concerns were identified with the peer reviewers assigned to this article; the investigation has concluded that the peer review process was compromised. We therefore can no longer trust the peer review process and the article is being retracted with the agreement of the Chief Editor.

The authors do not agree with the retraction.
